# Does bicompartmental knee arthroplasty hold an advantage over total knee arthroplasty? Systematic review and meta-analysis

**DOI:** 10.1051/sicotj/2021036

**Published:** 2021-07-09

**Authors:** Hany Elbardesy, Ahmed K. Awad, André McLeod, Samar Tarek Farahat, Somaya Zain Elabdeen Sayed, Shane Guerin, James Harty

**Affiliations:** 1 Department of Trauma and Orthopaedic, Cork University Hospital Wilton, Cork T12DFK4 Ireland; 2 School of Medicine, Ain-Shams University Cairo 11566 Egypt; 3 Faculty of Medicine, Menofia University Menofia 51132 Egypt; 4 Faculty of Medicine, Minia University Minia 61519 Egypt

**Keywords:** Uni knee, Patellofemoral arthroplasty, Total knee arthroplasty, Meta-analysis

## Abstract

*Introduction*: The role of bicompartmental knee arthroplasty (BKA) in the treatment of medial patellofemoral osteoarthritis (MPFOA) has been debated by orthopaedic surgeons for years. The BKA is a cruciate ligament retaining prosthesis designed to mimic the kinematics of the native knee that requires resurfacing of only two knee compartments. In this study, we aim to assess the patient recorded outcome measures (PROMs), range of motion (ROM), perioperative morbidity, and implant revision rate in patients undergoing BKA and compare them to those undergoing total knee arthroplasty (TKA) for bicompartmental knee osteoarthritis (OA). *Patients and methods:* We followed the Preferred Reporting Items for Systematic Reviews and Meta-analyses Statement (PRISMA). Articles from any country and written in any language were considered. We included all randomized control trials and retrospective cohort studies examining BKA versus TKA for bicompartmental knee OA. The primary outcome measure was knee society score (KSS) at one year and the secondary outcome measures were Oxford knee score (OKS) and short-form survey (SF-)12 at six and twelve months. *Results*: We included five studies in our meta-analysis. In terms of OKS, KSS, and SF-12, our meta-analysis suggests better short-term results for the TKA compared with the BKA. TKA was also associated with a shorter operative time and a lower revision rate. The BKA implant did however result in marginally less intraoperative blood loss and slightly better post-operative ROM. *Conclusions*: BKA did not prove to be an equivalent alternative to TKA in bicompartmental knee OA. It was associated with inferior KSS, OKS, and SF-12 at short-term follow-up and a higher revision rate.

## Introduction

Total knee arthroplasty (TKA) is a recognized treatment option for knee osteoarthritis representing 84.3% of the total number of knee arthroplasty reported by the Australian Orthopaedic Association National Joint Replacement Registry (AOANJRR) over the last 15 years. This is in comparison to partial knee arthroplasty which accounts for only 7.7% of this cohort [1]. The recorded survival rate at 25 years follow-up was 82% for TKAs compared with 70% for Uni-compartmental Knee Arthroplasty (UKA) [2]. A recent study showed that of the patients waiting for knee arthroplasty, 51% had medial compartment OA, 6.5% had lateral compartment OA, and 1.2% had patellofemoral osteoarthritis (PFOA). Tri-compartmental OA was found only in 16.7% and medial patellofemoral osteoarthritis (MPFOA), a combination of the medial compartment and patellofemoral OA, was found in 15.5% [3]. This suggests that a proportion of patients who receive TKAs are undergoing resurfacing of a non-arthritic lateral compartment with intact anterior cruciate ligament (ACL). In theory, these patients could be adequately managed with bicompartmental knee arthroplasty (BKA) resulting in reduced intra-operative blood loss and preserving the cruciate ligaments thus maintaining the natural kinematics of the native knee [4, 5]. Some authors have advocated for bi-compartmental knee replacement as it is associated with less blood loss, fewer side effects, and quicker rehabilitation than TKA [6]. As such, selective knee compartment replacement has been adopted by some researchers with promising outcomes [7, 8]. Bi-Compartmental OA (BCOA) was not traditionally recommended for UKA. Thus, BKA was advocated to be used in the treatment of BCOA [3, 7]. This meta-analysis aims to evaluate studies assessing Patient Recorded Outcome Measures (PROMs) for both BKA and TKA for the treatment of BCOA and to use a systematic approach to comparatively evaluate variables including operating time, postoperative Range of Motion (ROM), intraoperative blood loss, and revision rate.

## Materials and methods

**Figure 1:**
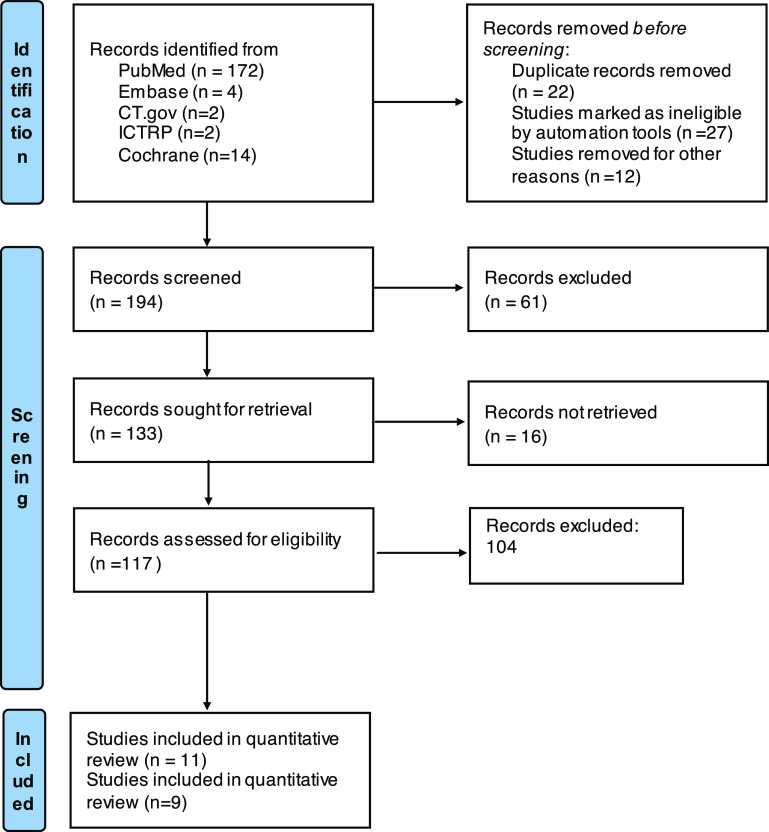
Preferred reporting items for systematic reviews and meta-analyses (PRISMA) flow chart.

We followed in this review both the Preferred Reporting Items for Systematic Reviews and Meta-analyses Statement (PRISMA), (Fig. 1), and the Cochrane Handbook for systematic reviews and meta-analysis [9]. We conducted an initial search using Web of Science, PubMed, EMBASE-OVID, Google Scholar, and Cochrane Library. We used the following keywords and their combinations: medial pivot, posterior stabilized, and total knee arthroplasty. Articles published up to March 2021 were included in our literature search and were limited to studies in human subjects published in any language. Additionally, we cross-referenced the bibliographies of retrieved articles and review papers to ensure that we captured all relevant studies.

### Study selection criteria

We included all comparative studies (retrospective/prospective cohorts, randomized clinical trials (RCTs)) involving patients undergoing unilateral or bilateral TKAs which were of MP or PS design, and where outcomes were compared between the two designs. We excluded cadaveric, in vitro, or single-arm studies. Conference abstracts, letters to the editor, reviews were also excluded.

### Data extraction and analysis

Five authors independently screened all titles and abstracts identified by the initial search to assess their eligibility for inclusion. We then did a full screening of each manuscript and conducted a final assessment of the eligibility for all included studies. The same reviewers performed the data extraction. Any discrepancies found after data collection were resolved by discussion between all reviewers. The collected information included first author, year, journal, country, level of evidence, study design, number of centers, study length, number of participants, age, gender, and Body Mass Index (BMI).

### Methodological quality assessment

We assessed the risk of bias for RCTs by using the Cochrane risk of bias criteria and the nonrandomized cohort studies using the Newcastle-Ottawa Scale [9, 10]. Five reviewers independently crossed-checked the quality of the included studies. Disagreements were resolved through discussion.

### Risk of Bias (ROB) assessment

**Figure 2:**
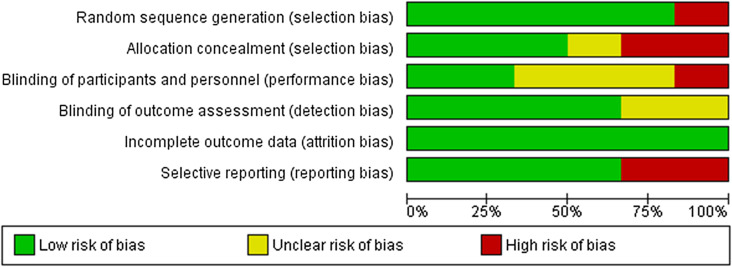
Risk of bias graph: review authors’ judgments about each risk of bias item presented as percentages across all included studies.

**Figure 3:**
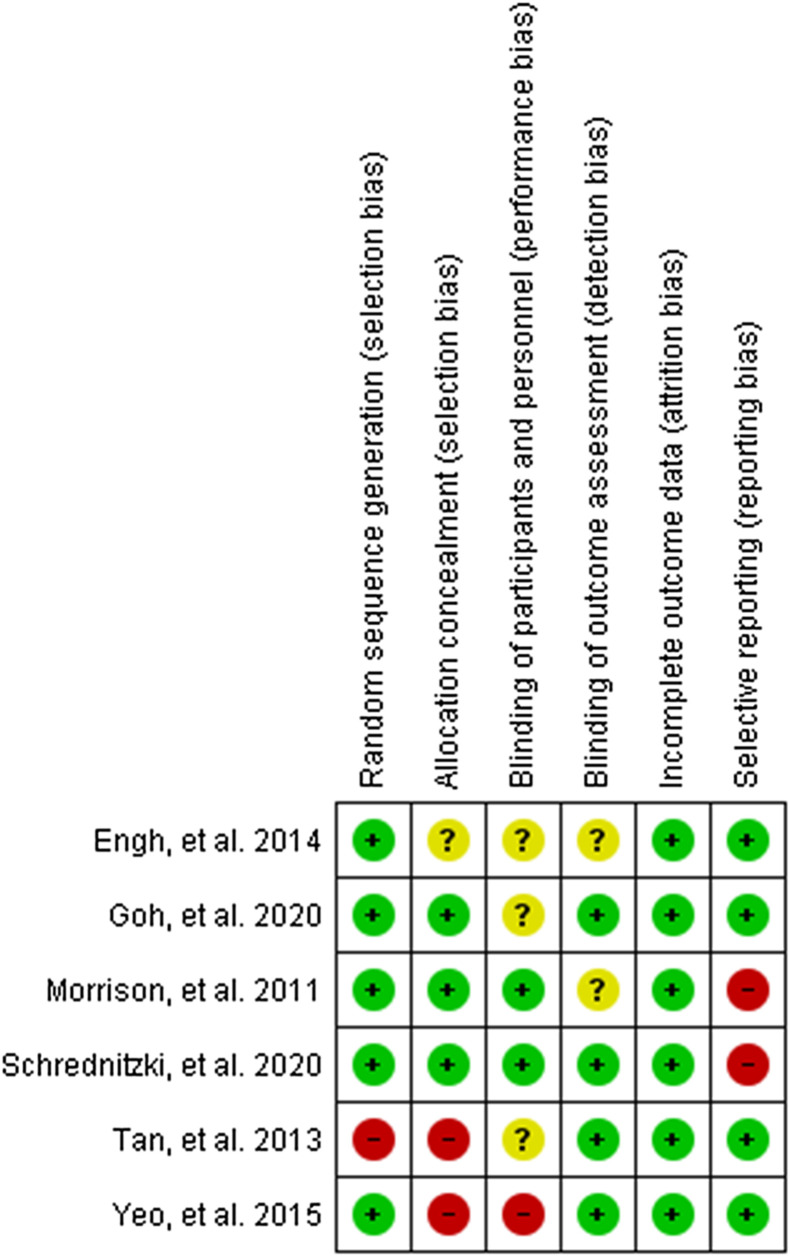
Risk of bias summary: review authors’ judgments about each risk of bias item for each included study.

Six RCTs were assessed for potential bias using the Cochrane risk of bias tool. A summary of the risk of bias is shown in (Figs. 2 and 3). Randomization and patient blinding were adequate in five studies [11–15], and unclear in the other one study [16]. A high risk of bias was not found in any of the six studies, yet some concerns were raised regarding one of the domains of all six studies. All the studies had clear judgment in at least one of the domains. Quality assessment of five non-randomized cohort studies using the Newcastle-Ottawa Scale showed that all studies were of high quality (Table 1).

**Table 1:** Newcastle-Ottawa Scale (NOS) for assessing the quality of observational studies.

Study	Selection				Comparability	Exposure			Total
	Representativeness of the exposed cohort	Selection of the non-exposed cohort	Ascertainment of exposure	Demonstration that outcome of interest was not present at start of study	Comparability of cohorts based on the design or analysis	Assessment of outcome	Was follow-up long enough for outcomes to occur	Adequacy of follow up of cohorts	Total number of stars
Tan et al. [22]	*	*	*	*	*	*	*	*	8
Siddharth et al. [21]	*	*	*	*	*	*	*	*	8
Biazzo et al. [23]	*	*	–	*	**	*	*	*	8
Chung and Min 27	*	*	*	*	*	*	*	*	8
Parratte et al. [16]	*	*	-	*	*	*	*	*	7
Confalonieri et al. [32]	–	*	*	*	*	*	*	*	7

### Outcome measures

We assessed three variables in our meta-analysis, Oxford Knee Score (OKS) [17], Knee Society Score (KSS) [18], and 12-Item Short Form Survey (SF-12) [19]. We examined four other variables in our systematic review including operating time, postoperative Range of Motion (ROM), intraoperative blood loss, and revision rate. Sufficient data were not available to conduct a meta-analysis on these variables.

### Statistical analysis

We conducted a statistical analysis by using Review Manager (RevMan), version 5.3 (The Nordic Cochrane Centre, The Cochrane Collaboration, 2009, Copenhagen, Denmark) [20]. Heterogeneity between studies was assessed by the *I*^2^ statistic and a *c*^2^ of <0.05 was used to define the significance of the heterogeneity among the included studies. Ranges of 0–24%, 25–74%, and 75–100% were used to define minor, moderate, and major heterogeneity respectively [9]. Mean differences and standard deviations (SDs) were used for continuous variables. We used the random-effects model in our meta-analysis. We illustrated the results using forest plots, which used a 95% confidence interval (CI) for each study and a cumulative weighted Mean Difference (MD) for all the included studies [9].

## Results

### Study characteristics

Our literature review returned 172 articles after the removal of duplicates. Title and abstract screening revealed 133 articles that were eligible for full-text screening. 121 articles were subsequently excluded for not meeting selection criteria leaving eleven articles that were included for qualitative review. Nine of these articles were included in the meta-analysis. A flow chart demonstrating the study selection process is provided (Fig. 1). Six studies were randomized clinical trials (RCTs), and five were retrospective cohort studies. A summary of the characteristics of included studies is provided (Table 2).

**Table 2:** Study characteristics.

Study	Year	Country	Journal	Study type	PROM	Revision Rate
Schrednitzki et al. [13]	2020	Germany	The journal of arthroplasty	RCT	KSS, OKS, and the University of California, Los Angeles scores, SF-12	1 at BKA group
Yeo et al. [12]	2015	Singapore	The knee	RCT	BKS, OKS, AKSS, pre and post-operative range of motionSF-12	1 at BKA group
Tan et al. [22]	2013	Singapore	*Journal of Orthopaedic Surgery*	RCT	KSS, WOMAC, and SF-36 scores, range of movement, Pain score (VAS)	0 at both groups
Siddharth et al. [21]	2013	Singapore	The journal of knee surgery	RCS	KSS-clinical, KSS-function, KSS-total, KOOS-pain, KOOS symptoms,KOOS-stiffness, and KOOS-ADL WOMAC pain	1 at BKA group
Morrison et al. [11]	2011	USA	The journal of arthroplasty	RCT	SF-12 and WOMAC	3 at BKA group
Engh et al. [14]	2014	USA	The journal of arthroplasty	RCT	KSS, OKS	3 at BKA group, and 1 at TKA group
Biazzo et al. [23]	2019	Italy	MUSCULOSKELETAL SURGERY	RCS	KSS	1 at TKA group
Chung and Min 27	2013	Korea	KSSTA	PCS	knee extensor and flexor torque, hamstring/Quadriceps (H/Q) ratio, knee position sense, and physical performance, proprioception	N/A
Parratte et al. [16]	2015	Belgium and France	Orthopaedics & Traumatology: Surgery & Research	RCS	KSS function, KSS knee, UCLA score.	N/A
Confalonieri et al. [32]	2009	Italy	Arch Orthop Trauma Surg	RCS	Post-op HKA angle, IKS score, FUNCT score, GIUM scoreWOMAC pain, function, and stiffness	N/A
Goh et al. [15]	2020	Singapore	The Knee	**RCT**	KSS function, KSS knee, OKS, SF36PCS, SF36 MCS, knee flexion	1 TKA group

Note: KSS: Knee society score, OKS: Oxford knee score, AKSS: American knee society score, SF-12: Short Form 12, WOMAC: Western Ontario and McMaster Universities Osteoarthritis Index assessments, RCT: randomized control study, RCS: retrospective cohort study, PCS prospective cohort study, KSSTA: Knee Surg Sports Traumatology Arthroscopy.

### Patient baseline characteristics

Our review included 561 knees (310 in BKA group, 251 in TKA group). The BKA group had an average age of 59.25 years (±6.25 years), of which 183 out of 310 patients (60.65%) were female, with an average body mass index of 28.37 kg/m^2^ (±3.32). The TKA cohort had a patient distribution with an average age of 62.69 years (±5.5 years), of which 167/251(66.53%) were female, with an average body mass index of 29.3 kg/m^2^ (±3.76). A summary of the patient demographics of included studies is provided (Table 3).

**Table 3:** Patient’s demographics.

Study	Centers	Total number	BKA	TKA	Gender female		Age (SD)		BMI (SD)		Follow-up	
					BKA	TKA	BKA	TKA	BKA	TKA	BKA	TKA
Schrednitzki et al. [13]	one center	80	40	40	20	20	65.25 (8.9)	63.55(6.6)	32.9(6.1)	34.7(6.5)	5 years	5 years
Yeo et al. [12]	one center	48	26	22	21	16	63.8 (8.03)	63.1 (7.3)	27.28(3.04)	28.15(4.52)	5 years	5 years
Tan et al. [22]	One center	27	15	12	8	9	52 (41–62)	60 (41–63)	26.0 (4.2)	28.3 (4.9)	20 months	20 months
Siddharth et al. [21]	one center	36	16	20	10	16	52.1 (6.4)	65.1 (7)	27.6 (4.4)	27.3 (3.8)	2 years	2 years
Morrison et al. [11]	one center	71	50	21	25	15	63.2 (11.5)	67.18 (9.5)	31.7 (7.7)	33.7 (8.6)	2 years	2 years
Engh et al. [14]	One center	75	50	25	25	12	60.3	58.3	28.8	30	2 years	2 years
Biazzo et al. [23]	One center	40	20	20	16	17	67.2	65	27.6	29.7	38 months	38 months
Chung and Min 27	One center	24	11	13	7	11	54.8 (5.6)	65.7 (6.7)	27 (2.8)	25.4 (2.5)	12 months	12 months
Parratte et al. [16]	Two center	68	34	34	21	21	61 (7)	61 (8)	27.5 (4)	27.5 (4.5)	2 years	2 years
Confalonieri et al. [32]	One center	44	22	22	14	14	60.4 (6.06)	60.7 (5.96)	N/A	N/A	48 months	48 months
Goh et al. [15]	One center	48	26	22	21	16	63.8 (8.03)	63.1 (7.34)	27.28 (3.04)	28.15 (4.52)	10 years	10 years

SD: standard deviation.

### Meta-analysis

Our meta-analysis comparatively assessed the KSS, OKS, and SF-12 scores at six months and one-year follow-up.

#### Oxford knee score

**Figure 4:**
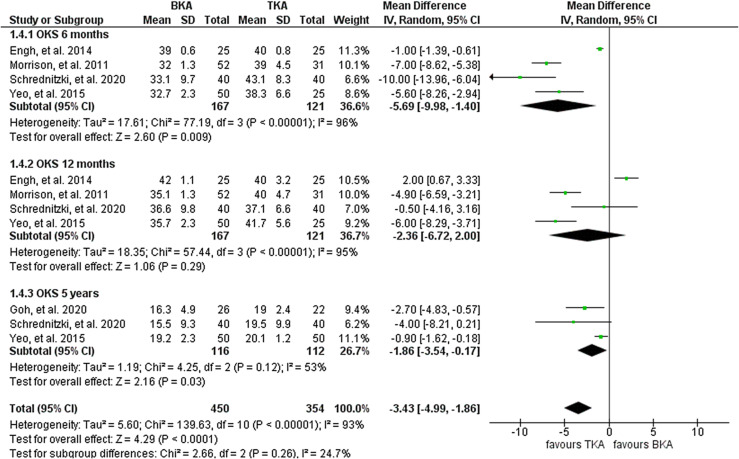
Forest plot of comparison: BKA vs TKA, outcome: OKS after 6, 12 months, and 5 years.

Overall, four studies including 288 knees (167 BKA; 121 TKA) reported on OKS after six months and one year. They reported a significantly higher OKS for the TKA cohort at six months and marginal improvement at one-year follow-up. Three studies reported on OKS after 5 years encompassing 219 knees (112 BKA and 107 TKA). They reported a significantly superior OKS for the TKA cohort. Heterogeneity analysis demonstrated high statistical evidence for variation within the studies (*I*^2^ = 93%). The cumulative MD was significant at −3.43 (95% CI, −5.0−1.86; *P* < 0.001) (Fig. 4).

#### Knee society score

**Figure 5:**
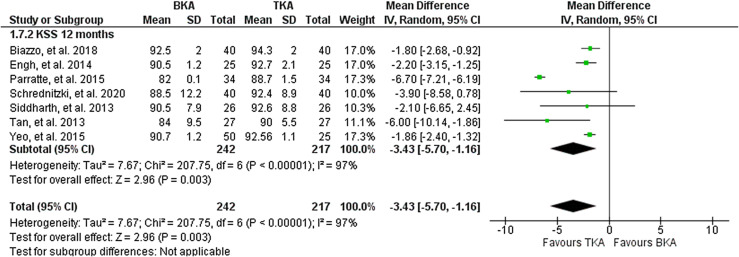
Forest plot of comparison: BKA vs TKA, outcome: KSS after 12 months.

Overall, seven studies including 459 knees reported on postoperative KSS score after one year. They reported a significantly better KSS for the TKA cohort. Heterogeneity analysis demonstrated high statistical evidence for heterogeneity (*I*^2^ = 97%). The cumulative MD was significant at −3.43 (95% CI, − 5.70 − 1.16; *P* < 0.005, Fig. 5).

#### SF-12 score

**Figure 6:**
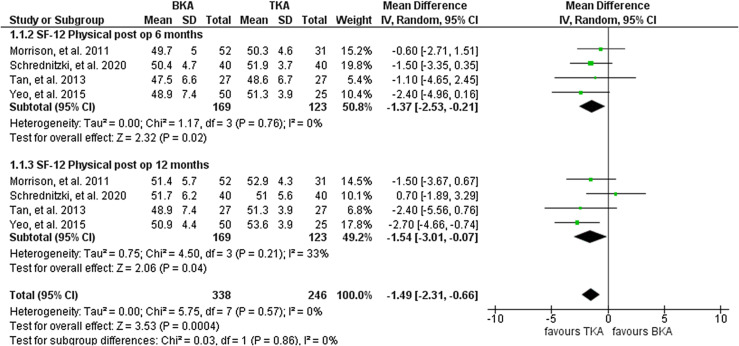
Forest plot of comparison: BKA vs TKA, outcome: SF-12 after 6 and 12 months.

Four studies including 292 knees (169 BKA; 123TKA) reported on SF-12 after six months and one year. They reported significantly higher SF-12 scores for the TKA cohort at six and twelve months. The heterogeneity analysis demonstrated no statistical evidence for variation within the study (*I*^2^ = 0%). The cumulative MD was significant at −1.49 (95% CI, −2.31 – 0.07; *P* < 0.001) (Fig. 6).

### Systematic review

#### ROM

ROM was marginally greater in the BCA cohort [13, 16, 21, 22]. One study reported an improvement in the degree of knee flexion [16].

#### Operative time

The mean time for surgery was significantly longer for BCA (73.5 ± 9.9 min) compared to TKA (58.8 ± 12.8 min; *P* < 0.05) [13, 22, 23].

#### Blood loss

A higher average blood loss was reported in the BCA group in only one study [13]. It was reported as being significantly less than the TKA group in four studies (*P* < 0.05) [12, 15, 22, 23].

#### Revision rate

About the postoperative revision rate, the BKA cohort reported 9 cases out of 310 (2.9%) after a mean follow-up of 45 months. However, the TKA reported only 3 revisions out of 251 (1.2%) within the same time frame.

## Discussion

The most important findings in this meta-analysis were that the post-operative KSS, OKS, and SF-12 were significantly higher in the TKA cohort compared with the BKA. However, the BKA showed less intraoperative bleeding and a slight but insignificant superiority in terms of post-operative ROM. To our knowledge, only one meta-analysis (MA) [24], has been published examining the same topic with the inclusion of seven studies in their qualitative review and two studies in the MA. The included studies in that article were published up to September 2015. Five studies examining this topic have been published since that date and are all included in our study. This may explain the contradiction in the results between the two articles. The survivorship of BKAs is debatable. One author reported 80% survivorship of BKA at 17 years [25], while another study including only nine patients showed 100% survival of the BKA at 12 years with very good functional outcomes [26]. Parratte et al. [25], examined 71 patients with BKAs and reported a 54% survival rate at 17 years to follow up. This is more than five times the revision rate reported for the TKA in another study which was 9.0% at 19 years [1]. Of note, the author also raised concerns regarding the design of the BKA prosthesis and it is technically demanding implantation when used for both unicompartmental and patellofemoral osteoarthritis [25].

Chung and Min [27] compared the quadriceps muscle strength between BKA and TKA patients. They could not detect any significant difference between the two cohorts despite the theoretical advantage for the BKA due to the preservation of cruciate ligaments and greater bone stock. Benazzo et al. [28], reported a revision rate of 10% (3 out 30) for BKA, two of them were for patella resurfacing and the other one was due to aseptic loosening all within 5 years. This was more than quadruple the revision rate for the TKA (2.23%) within the same time frame [29]. Another study reported revision of 2 cases out of 41 (4.8%) for BKA at 6 years follow up, with the main reason for revision being aseptic loosening and knee pain [30]. Theoretically, the BKA mimics the native knee kinematics by preserving the cruciate ligaments and good bone stock [31–36]. However, the progression of Osteo Arthritis (OA) in the third compartment raises a major concern for BKA [26]. After a mean of 11.8 (±5.4) years follow-up, Heyse et al. [26], reported progression of OA within the third compartment in about 55% of patients (five out of nine) who had undergone BKA. A long-term study examined both the BKA and The TKA for 10 years follow up and did not report any significant difference between the two cohorts, however, the number of patients included in each group was small (26 and 22 respectively) [15]. Moreover, the high percentage of loss to follow up (15% and 22.7% respectively) means we must take these results with caution. The marginal advantage in the postoperative ROM for the BKA does not make up for the complexity of the surgical technique and the higher revision rate and inferior PROMS reported for it. Moreover, the less intraoperative bleeding for BKA is outweighed by the shorter operative time reported for the TKA. Overall, the BKA did not show any significant advantages over the TKA in the context of OA and thus we recommend against it.

### Study strengths and limitations

One of the strengths of our study is the large number of studies included in our analysis (11 studies). As well as this a significant portion of our included studies is modern, with all included studies being published between 2009 and 2020. In terms of study limitations, the data used in this study was obtained from several studies reporting the ROM and PROMs between the BKA and TKA. The techniques and materials used in these studies were similar but not identical. Another limitation is the inclusion of five retrospective studies in the meta-analysis. The observational patterns associated with retrospective cohort studies are more susceptible to bias in data collection. Another source of limitation was the lack of long-term follow-up. While the studies included reported scores for up to 5 years follow-up, there is a paucity of data beyond this. We would recommend more RCTs with a long-term follow-up period examining these two prosthetic designs.

## Conclusion

In terms of KSS, OKS, and SF-12, this meta-analysis suggests better short-term results for the TKA compared with the BKA. The TKA was also associated with a shorter operative time and a revision rate similar to BKA at short-term follow-up. The BKA implant showed marginally lower intraoperative blood loss and slightly better ROM. However, it also reported a relatively high failure rate in mid and long-term follow-up in comparison to the TKA that led us to advise against its use.

## Contributors

All authors contributed to the study’s conception and design. Conceptualization: [Hany Elbardesy and Ahmed K. Awad], Methodology: [Shane Guerin and Somaya Zain Elabdeen Sayed], Formal analysis and investigation: [Hany Elbardesy and Ahmed K. Awad], Writing – original draft preparation: [Hany Elbardesy, Ahmed K. Awad, Samar Tarek Farahat, and André McLeod]; Writing – review and editing: [Hany Elbardesy and James Harty], Supervision: [James Harty and Shane Guerin] and all authors commented on previous versions of the manuscript. All authors read and approved the final manuscript.

## Compliance with ethical standards

*Conflict of Interest*: The authors declare that they have no conflict of interest.

*Funding*: There is no funding source.

*Ethical approval*: This article does not contain any studies with human participants or animals performed by any of the authors.

*Informed consent*: not applicable.
